# Non-Linear Optical Microscopy Sheds Light on Cardiovascular Disease

**DOI:** 10.1371/journal.pone.0056136

**Published:** 2013-02-07

**Authors:** Valentina Caorsi, Christopher Toepfer, Markus B. Sikkel, Alexander R. Lyon, Ken MacLeod, Mike A. Ferenczi

**Affiliations:** 1 Molecular Medicine, National Heart and Lung Institute, Imperial College London, London, United Kingdom; 2 Myocardial Function, National Heart and Lung Institute, Imperial College London, London, United Kingdom; 3 Cardiovascular Biomedical Research Unit, Royal Brompton Hospital, London, United Kingdom; 4 Lee Kong Chian School of Medicine, Nanyang Technological University, Singapore, Singapore; University of Minnesota, United States of America

## Abstract

Many cardiac diseases have been associated with increased fibrosis and changes in the organization of fibrillar collagen. The degree of fibrosis is routinely analyzed with invasive histological and immunohistochemical methods, giving a limited and qualitative understanding of the tissue's morphological adaptation to disease. Our aim is to quantitatively evaluate the increase in fibrosis by three-dimensional imaging of the collagen network in the myocardium using the non-linear optical microscopy techniques Two-Photon Excitation microscopy (TPE) and Second Harmonic signal Generation (SHG). No sample staining is needed because numerous endogenous fluorophores are excited by a two-photon mechanism and highly non-centrosymmetric structures such as collagen generate strong second harmonic signals. We propose for the first time a 3D quantitative analysis to carefully evaluate the increased fibrosis in tissue from a rat model of heart failure post myocardial infarction. We show how to measure changes in fibrosis from the backward SHG (B_SHG_) alone, as only backward-propagating SHG is accessible for true *in vivo* applications. A 5-fold increase in collagen I fibrosis is detected in the remote surviving myocardium measured 20 weeks after infarction. The spatial distribution is also shown to change markedly, providing insight into the morphology of disease progression.

## Introduction

The importance of increased fibrosis in heart pathology and dysfunction has been increasingly noted in diseases including dilated, ischaemic and hypertrophic cardiomyopathies. In healthy tissue the extracellular fibrillar collagen forms a dynamic scaffold with important functions for tissue integrity and efficiency of systolic contraction and diastolic relaxation [1]. Increased collagen deposition in reactive fibrosis leads to altered myocardial function, specifically reducing tissue compliance and impairing both contraction and relaxation [2]. The degree of fibrosis is routinely analyzed in experimental models, and at *post mortem* in human hearts, with histological and immunohistochemical methods. These require invasive extraction of tissue samples, thin slicing by microtomes (1–10 µm sections), embedding, fixation, and staining procedure [3], [4]. Although these methods allow collagen to be clearly distinguished, they report from a thin layer of the sample under investigation, giving a limited and qualitative understanding of the tissue's morphological adaptation to disease. Furthermore myocardial tissue acquisition in the clinic is limited, and sample processing can introduce artifacts thus limiting its use [5]. Three-dimensional imaging of the collagen network in the myocardium is required to quantitatively assess the status of fibrosis *in vivo* and hence understand how changes in its production affect the progression of cardiac dysfunction. Recently non-linear optical microscopy has been shown to be a powerful tool for non-invasive imaging of thick specimens [6], [7]. In particular two-photon excitation (TPE) and second harmonic generation (SHG), arising from the non linear interaction of intense coherent optical radiation with matter, have been cleverly exploited to obtain information otherwise inaccessible by conventional microscopy [8]. TPE fluorescence and SHG are particularly suitable for imaging thick samples as both processes intrinsically give optical sectioning capability. Furthermore the infrared excitation light used to induce the two-photon excitation process can enter deeper into the sample because scattering is reduced [9], [10]. There is no need for sample staining, thus removing major sources of artifact: SHG arises from a particular type of molecule lacking a center of symmetry (as collagen, microtubules, myosin) and numerous endogenous fluorophores can be excited by a two-photon mechanism [11]–[14]. Collagen is particularly suitable for SHG collection as the inherent chirality of the helices increases the overall asymmetry thus increasing the second order response. In particular collagen type I and II have been shown to experimentally produce sufficient SHG signals, while collagen type III has a much weaker response [6]. The combination of these two techniques has already provided a new tool for diagnosis of many diseases characterized by defects or changes in the assembly of collagen in a variety of tissues [15], [16], [17]. However, to our knowledge, only qualitative or semi-quantitative analysis has been performed in cardiac disease [18], [19], [20].

Our aim is to combine the use of TPE (to visualize endogenous fluorophores like elastin and myocyte proteins) with the simultaneous collection of SHG from collagen fibrils (type I) in thick cardiac tissues to quantitatively evaluate the increase in fibrosis by analyzing the backward SHG (B_SHG_) alone, as only backward-propagating SHG is accessible for future *in vivo* applications. In particular we propose a 3D intensity-based and volumetric method which can be easily implemented in any laser scanning microscope equipped with multiphoton excitation with no modifications to the light path, thus enabling and enhancing its applicability. To validate the proposed methodology we evaluate collagen presence in the non-infarcted, viable portion of the left ventricle in a rat model of myocardial infarction [21]. In this model the anterior wall becomes scarred and clearly contains substantial collagen. The remodeling process that occurs in the remainder of the left ventricular wall includes interstitial collagen deposition in non-infarcted tissue, contributing to the decline in the function of this surviving muscle [22], [23], [24]. We demonstrate a 5-fold increase in collagen fibrosis 20 weeks after infarction. The spatial distribution within the observed volume also changes markedly, providing insight into the morphology of disease progression. The results obtained show how a quantitative evaluation of fibrillar collagen in fresh, untreated tissues is possible, giving information otherwise inaccessible with routine histology methods, thus shedding light on myocardium morphological adaptations to disease *in vivo*.

## Materials and Methods

### Animal model for heart failure

All animal surgical procedures and perioperative management were carried out in accordance with the United Kingdom Home Office Animals (Scientific Procedures) Act 1986, which conforms to the Guide for the Care and Use of Laboratory Animals published by the US National Institutes of Health under assurance number A5634-01 (NIH publication No. 85-23, revised 1996). The protocol was approved by the Imperial College Ethical Review Committee (PPL: 70/6568 and 70/7399). Adult male Sprague-Dawley rats (250–300 g) underwent proximal left anterior descending coronary artery ligation to induce chronic myocardial infarction [21]. Anaesthesia was induced by administration of 5% isoflurane for induction reducing to 2% isoflurane once intubated and ventilated. It was ensured that pain reflexes were absent prior to an incision being made. Pre-operatively, buprenorphine was administered subcutaneously at a dose of 0.05 mg/kg to ensure adequate analgesia in addition to anaesthesia. Further doses of buprenorphine were administered as required post-operatively if there was any sign of distress. Additionally enrofloxacin (5 mg/kg) and 0.9% saline (10 ml/kg) were administered pre-operatively. Rats were sacrificed by cervical dislocation following brief exposure to 5% isoflurane until righting reflex was lost. Age matched rats which had not undergone surgery were used as controls. Animals were sacrificed at 20 weeks post-surgical intervention by which time a severe heart-failure phenotype has developed. Rapid explantation of rat hearts was performed and hearts were immediately massaged in a modified oxygenated and iced Krebs-Henseleit (KH) solution (composition in mM: 119 NaCl, 4.7 KCl, 0.94 MgSO4, 1 CaCl2, 1.2 KH2PO4, 25 NaHCO3, 11.5 glucose) containing 40 mM BDM and Heparin. Hearts were cannulated through the aorta for retrograde perfusion by Langendorff apparatus with oxygenated KH solution [25]. Trabecular preparations (100–120 µm in diameter and 1–2 mm in length) were excised from the inner wall of the left ventricle, T-clipped and permeabilised in a Relaxing solution (composition in mM: 100 TES, 7.8 MgCl2, 5.7 Na2ATP, 25 EGTA, 21.2 Na2CP, 20 GLH, pH 7.1 at 20°C and ionic strength 200 mM) containing 2% Triton for 30 minutes, and stored at −20°C overnight in a relaxing solution containing 50% glycerol for use the following day. All the measurements were performed in relaxing solution which mimics the relaxed state in muscle and tissues with 0Ca and high ATP (zero calcium ensures no activation and high ATP ensures no rigor contractions). The effect of relaxing solution on the B_SHG_ intensity is addressed in the section *Maximizing Backward Collection* where a comparison with solutions at different NaCl concentrations is carried out.

### Histology staining

For histological analysis, left ventricular tissues were washed in sterile phosphate-buffered saline, fixed in 4% paraformaldehyde, processed for paraffin embedding. 4 µm thick sections were stained with picrosirus red to highlight interstitial collagen [26].

### Control samples: rat tail samples and skeletal muscle fibres

As controls, rat tail (∼100% collagen) and permeabilised skeletal muscle fibres (∼0% collagen) were used. Rat tail sections were dissected in relaxing solution and collagenous strands were isolated, T-clipped and stored in relax and glycerol solution at −20°C. Skeletal muscle fibers were isolated from the psoas muscle of adult male New Zealand white rabbits. Bundles were removed and permeabilized as described previously [27]. Bundles were stored for up to 8 weeks in relax and glycerol solutions at −20°C. Single muscle fibers from bundles were isolated 1–2 mm in length and T-clipped as previously described [28].

### Experimental microscope set-up and Imaging acquisition setting

All samples were mounted in relaxing solution between hooks on a custom-made set-up optimized for fitting the microscope stage. Two-photon excitation and SHG imaging was performed using a Leica TCS SP5 upright laser scanning system (Leica Microsystem), coupled to a titanium∶sapphire laser (Spectraphysics Mai Tai 690–1020 nm, 90 MHz; Spectra-Physics, Santa Clara, CA). All imaging was performed with an excitation wavelength of 900 nm (except for the spectra collection) with an average power of ∼10–12 mW (except for the power calibration measurements) at the specimen using a long working distance water immersion objective 25×0.9NA. Typical acquisition parameters are the following: 512×512 pixels, zoom 2.5, pixel size 0.53×0.53 µm, Kalman average of four images, pixel dwell time 18.75 µs, z-step size 0.4 µm. The SHG was collected in the backward direction by selecting the 440–460 nm range while autofluorescence light was collected in the 500–700 nm range. Typical z-stack of 100–150 µm have been acquired simultaneously collecting B_SHG_ and TPE autofluorescence. Lambda stacks for spectral analysis were collected from 390 nm to 700 nm (10 nm increments) exciting at different two-photon excitation wavelengths from 800 nm to 940 nm (20 nm increments). B_SHG_ versus excitation power were performed by varying the neutral filter at the laser output to increase the excitation power: power was measured after the objective (directly onto the samples) with a power meter (Thorlabs), ranging from 10 mW up to 70 mW. Forward vs Backward SHG measurements were performed by collecting 3D sectioning at different NaCl concentrations (0, 25 mM, 50 mM, 100 mM, 200 mM) compared to relaxing solution. Polarization measurements were performed by rotating the stage in 10degree increments. Intensity and volumetric analysis are performed on SHG and autofluorescence stacks using a custom-designed macro within the Java-based program ImageJ (http://rsb.info.nih.gov/ij/).

### Statistical analysis

In this study 16 animals (8MI and 8AMC) were used. From each animal 2–3 trabecular preparations were excised for imaging analysis. For each trabecula, four z-stacks (each nearly 100 µm depth) were collected in different areas (248 µm×248 µm) of the sample in order to control for inter sample variability. Both the intensity and the volume analysis are presented as mean and standard deviation.

## Results

### Collected light characterisation

**Figure 1:**
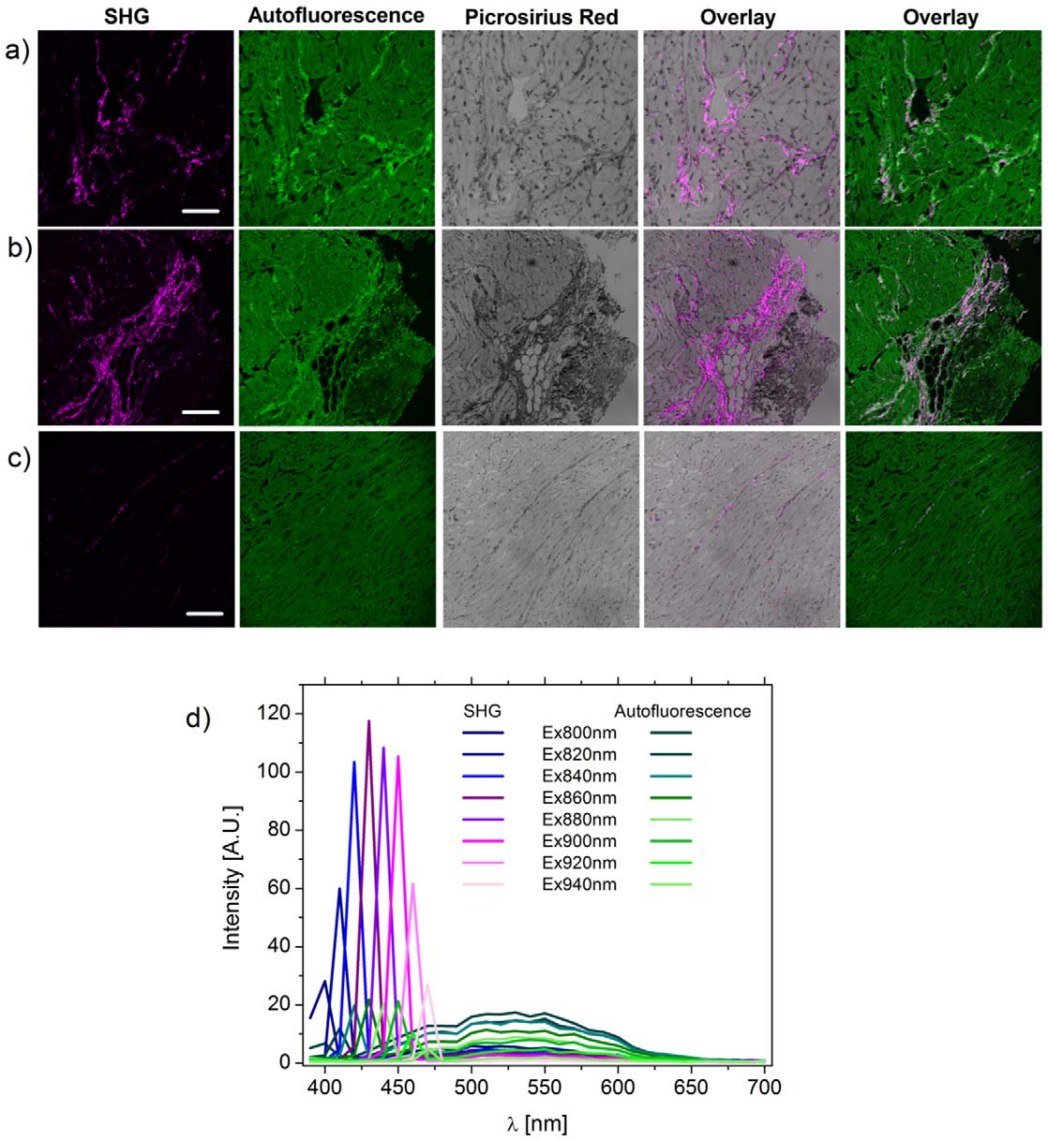
B_SHG_ and autofluorescence characterisation. Standard histology sections are stained with picrosirius red to highlight collagen in tissues from myocardial infarction rat model (a and b), from age-match control in c, showing the better signal to noise ratio for SHG light in discriminating collagen. In d, the spectral characterization is presented to demonstrate that the collected signals are SHG (magenta) by observing the shift in peak intensity with that of the excitation wavelength and autofluorescence (green). Scale bar 50 µm in a, 100 µm in b and c.

Histological sections of left ventricular tissues stained with Picrosirius Red (PR) are used to determine the biological origin of the SHG and autofluorescence signal collection. As shown in Fig. 1, (a and b from myocardial infarction tissues; c from age-matched control) SHG light discriminates collagen I with a better signal to noise ratio than PR, while autofluorescence highlights elastin and other components in the myocytes. It is worth underlining that Picrosirius Red stains both collagen type I and type III, without discriminating between the two and it is affected by unspecific binding; SHG is mainly due to type I as type III signal is probably too weak to be detected in the light collection arrangement used here. Overall these images show qualitative similarity between the two methods, with some mismatches. Nonetheless the SHG images show higher contrast and better specificity to detect collagen than PR. To demonstrate the physical origin of the signal, emission spectra are collected at different excitation wavelengths to distinguish B_SHG_ from autofluorescence. As shown in Fig. 1d, B_SHG_ peaks at exactly half the excitation wavelength, as expected from theory, correspondingly shifting with it, while autofluorescence spectra neither change shape or shift, showing a weaker intensity in a much broader λ-range. This spectral analysis represents the identity card of the collected light allowing for optimisation of excitation-emission settings. In experiments shown here, 900 nm excitation is a convenient compromise between B_SHG_ intensity, low autofluorescence (but still useful to outline the sample volume), penetration depth and Ti-sapphire laser performance.

### Calibration on rat tail samples and permeabilised skeletal muscle fibres:

**Figure 2:**
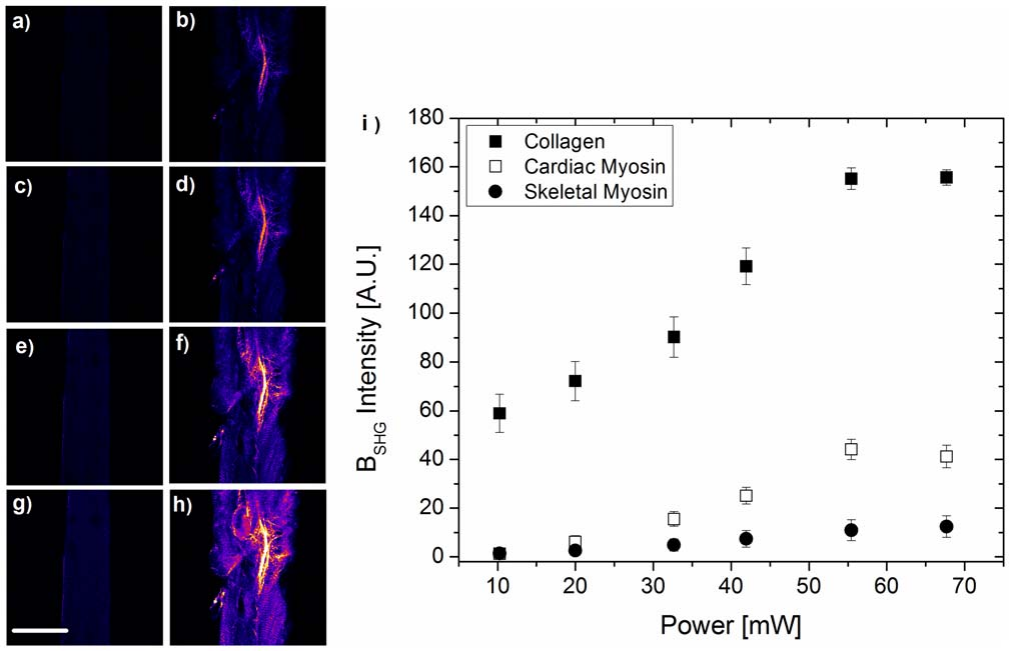
Myosin-B_SHG_ detection. Skeletal fibres (a, c, e, g, circle symbols in the graph) and cardiac trabeculae (b, d, f, h, squared symbols in the graph) imaged at increasing excitation power ranging from 10 mW up to 70 mW at 900 nm. In i), filled squared symbols are related to collagen-B_SHG_, while open squared symbols are related to myosin-B_SHG_, obtained from analyzing region of interest showing only collagen fibrosis or sarcomeric proteins respectively, as these are well spatially separated. By utilizing low power (10–20 mW) myosin-B_SHG_ can be neglected, thus ensuring the collection of only collagen-B_SHG_. Scale bar 100 µm.

**Figure 3:**
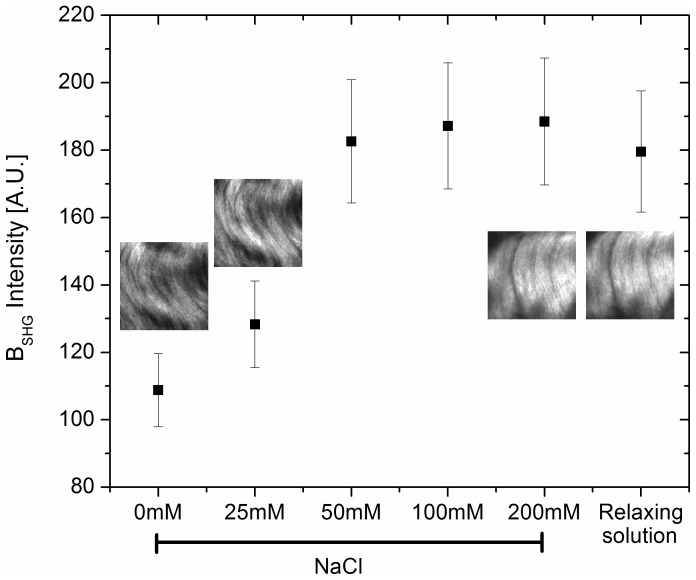
B_SHG_ dependence on ionic strength. B_SHG_ from rat tail samples has been collected at different NaCl concentrations compared to relaxing solution. From 50 mM NaCl the B_SHG_ reaches a plateau. Measurements performed in relaxing solution fall in the same range of intensity ensuring that in the present experimental conditions we maximize the backward light collection. Images above symbols are representative for that concentration.

**Figure 4:**
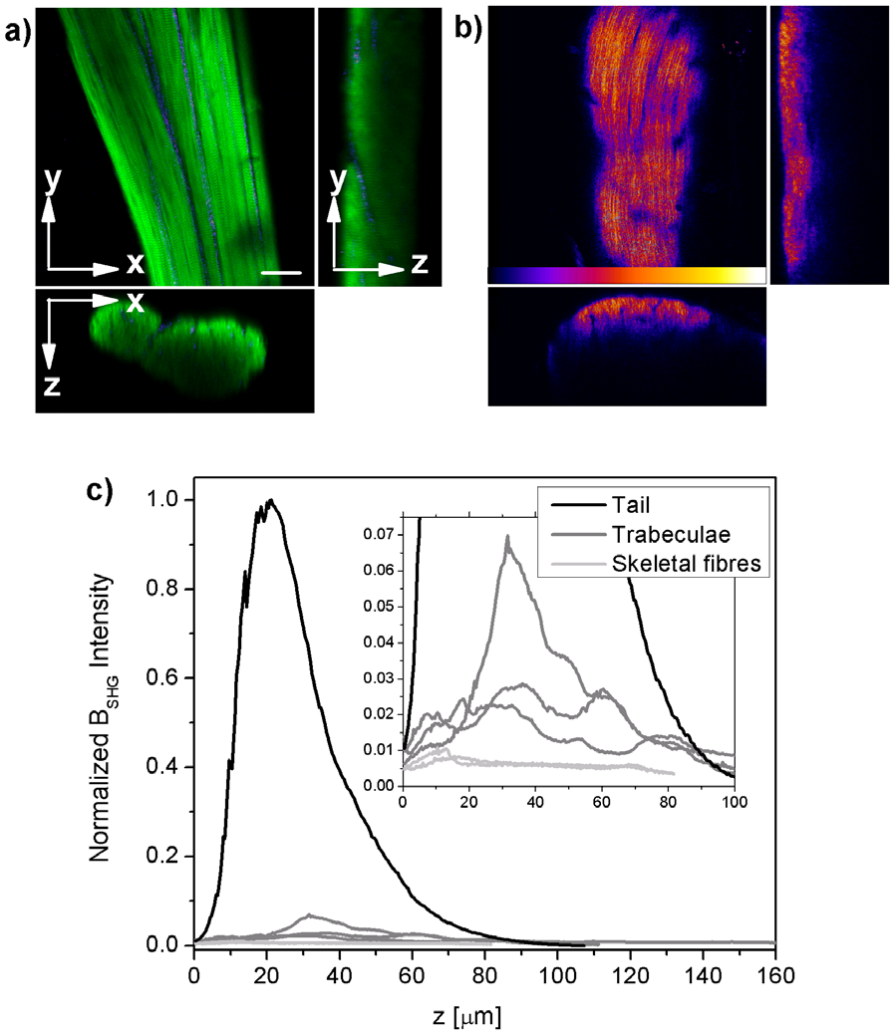
Upper and lower B_SHG_ detection range. Representative 3D optical sections (xy and orthogonal view xz, yz) of a trabecula in a) and a rat tail in b); green autofluorescence, fire color scale B_SHG_. Scale bar, 25 µm. In c) B_SHG_ intensity versus sample depth is presented for rat tail samples (black), cardiac trabeculae (dark grey) and permeabilised skeletal fibres (light grey). Intensity profiles have been normalized to the rat tail samples as they represent the upper bound of B_SHG_ detection. On the contrary permeabilised skeletal fibres show virtually no B_SHG_ (2%) thus representing the lower bound of detection. Healthy cardiac samples show a low but measurable B_SHG_, thus ensuring a large range of detection for possible increases in collagen content in diseased samples.

**Figure 5:**
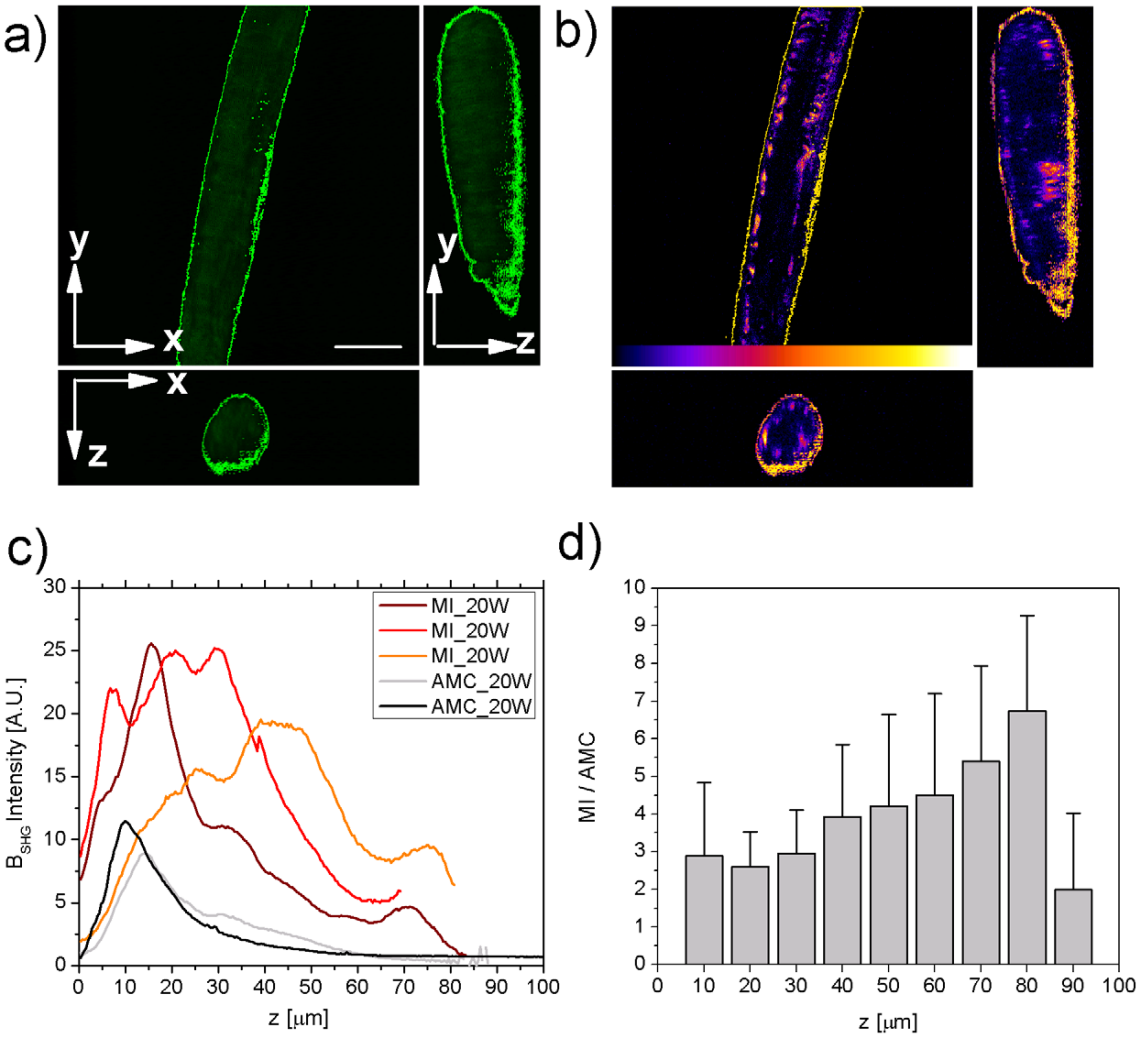
Intensity Analysis. 3D (shown as xy view and orthogonal views xz, yz) autofluorescence a) and B_SHG_ b) from a MI sample. The region of interest selected by thresholding the autofluorescence to define the sample edges is highlighted in green in a); the very same region is applied to the B_SHG_ images, highlighted in yellow in b); c) intensity profile along the thickness of the sample, MI (dark red, red, orange), AMC black and grey; d) Average intensity increase (MI/AMC) obtained by integrating B_SHG_ intensity over 10 µm z-steps.

1. **Excluding myosin-SHG.** In biological tissue, SHG has been shown to arise from collagen, microtubules as well as myosin [6], [13], [29], [30]. In order to assess changes in collagen deposits induced by cardiac disease, measurements rely on a signal arising solely from collagen, collagen-SHG. Determination of the origin of SHG was carried out by varying the excitation power of laser illumination on skeletal muscle fibres (Fig. 2 a, c, e, g) and cardiac tissue (Fig. 2 b, d, f, h). The B_SHG_ signal generated in Type II skeletal muscle fibres arises only from myosin while the one generated in cardiac tissues arises from both myosin and collagen: in this case, despite both collagen-SHG and myosin-SHG generate light at the same wavelength, the different localization within the muscle tissue allows for discriminating the two signals. In Fig. 2i, it can be seen that B_SHG_ generation by myosin requires very much higher laser intensity at 900 nm than collagen. Other excitation wavelengths were tested at increasing powers inducing a similar response (data not shown). In the power range 10 to 20 mW, the myosin-SHG signal can be ignored as it contributes to less than 5% of the total with a good signal to noise ratio for the collagen-SHG collection. All the measurements on infarcted and healthy cardiac tissues were performed at 10–12 mW, thus also limiting the induction of photodamage.
2. **Maximising Backward Collection.** While fluorescence is isotropic and hence emitted in all directions, SHG is anisotropic because of phase matching considerations and hence highly directional [31]. It is primarily forward directed, but imperfect phase matching causes SHG emission from tissues to have a distribution of components emitted forward (F) and backward (B) [14], [32]. As Williams et al. (2005) showed in addressing the origin of SHG from collagen [13], the directionality is highly dependent on the ionic strength of the solution in which the sample is analyzed. In particular they showed that at a physiological level of extracellular NaCl the F/B ratio decreases. We repeated this type of experiment by acquiring only B_SHG_ on rat tail fibrils at different NaCl concentrations in comparison with the relaxing solution used to image fresh cardiac tissues, at an ionic strength comparable to physiological saline concentrations (see materials for exact composition). Fig. 3 clearly shows the increase of B_SHG_ with increasing NaCl concentration, reaching saturation above 50 mM NaCl; measurements performed in relaxing solution were also at the plateau, demonstrating that in our experimental conditions, we maximize the B_SHG_ collection. It is worth noting however that the B_SHG_ we refer to, collected in the epi-mode, is partly due to true backward-SHG and partly due to back-scattered forward-SHG. This means that even though the truly backward SHG light intensity is usually quite small, with the contribution from the back-scatter a measurable SHG signal is obtained in tissue. Optimizing the ionic strength of the solution further maximizes the signal collection.
3. **Polarization effect.** SHG intensity is dependent on the angle between the collagen fibrils and the polarization of incident light [33]. Calibration measurements were performed on rat tail samples to evaluate the dependence on polarization angle by rotating the microscope stage in 10 degree steps (see Figure S1). The SHG intensity variation measured at different polarization directions is in agreement with the literature [29], [34]. We experimentally observed a small intensity modulation (nearly 6%) compared to previously published values [35], [36] because the polarization status at the focus is elliptical and the tail samples contain fibrils with different orientations, thus flattening the response. It was necessary to determine the role of polarization in our instrument to ensure that in our experimental conditions the polarization direction didn't affect the evaluation of the increase in B_SHG_ seen in MI samples compared to AMC. As an added precaution, the infarcted or healthy samples were imaged in the same orientation under the microscope. The experimental observation suggests that collagen fibres in both healthy and in diseased samples are mainly parallel to the main muscle axis. It is worth noting that increased fibrosis in diseased samples could lead to the appearance of collagen fibres which are not parallel to the main muscle axis, in the worse case perpendicular to it. In this case the B_SHG_ intensity evaluated would be underestimated by 6%. The use of circularly-polarized light would eliminate polarization-induced intensity differences.
4. **Detection Range.** Measurements to determine the B_SHG_ detection range were performed on rat tail samples, skeletal muscle fibres and cardiac trabeculae. We used rat tail samples as positive control because they are mainly composed of collagen type I, and single permeabilised skeletal muscle fibres as negative control because the permeabilisation process removes the outer membrane and the thin collagen sheath surrounding it. Fig. 4 shows the intensity of B_SHG_ collected for rat tail (black), three trabeculae (dark grey) and two skeletal fibres (light grey), normalized to the tail B_SHG_ intensity. The rat tail B_SHG_ response represents indeed the upper bound of detection; permeabilised skeletal fibres show little collagen and define the lower limit of detection (1–2% of rat tail signal); cardiac trabeculae from healthy hearts show a small but detectable intensity (5–6%) of B_SHG_ with respect to the tail, thus ensuring a maximal range (95%) allowance for the increased fibrosis expected in samples from infarcted hearts.
5. **Penetration Depth.** SHG intensity is limited by penetration depth. Depth attenuation is ruled by bulk optical properties: absorption coefficient, scattering, refractive index, etc. Experimentally the excitation light at the range of wavelengths used here (800–950 nm) is limited to 200–300 µm depth, depending on the tissue turbidity and scatterers distribution. The backward light collected (B_SHG_ convolved with scattering) rapidly decays within the first 100 µm as shown in Fig. 4 for rat tail segments. All the measurements were performed on trabecular preparations (the same muscle tissue) of similar thickness (in the range of 100–120 µm), so that similar scattering and attenuation effects are expected for the test and control trabeculae. In order to improve the penetration depth, clearing agents [32], [37] as well as adaptive optics [38], [39] could be employed. A 50% glycerol treatment has been already used in the sample preparation, allowing for a maximum penetration depth of nearly 100 µm, as shown in Fig. 4 and Fig. 5. Future measurements of the depth-dependent SHG directionality and attenuation responses in conjunction with simulations based on the bulk optical parameters [17] could refine our quantitative analysis.

### Quantifying disease by Intensity Analysis

To quantitatively analyze the increase in fibrosis, optical sectioning measurements were performed on non-infarcted trabeculae extracted from rats sacrificed 20 weeks following myocardial infarction (8MI samples) in comparison with age-match controls (8AMC samples), by simultaneously acquiring B_SHG_ arising from collagen and autofluorescence from elastin and other myocyte proteins. The autofluorescence image is used to define the edges of the sample, by thresholding the autofluorescence intensity as depicted in Fig. 5a. The same region of interest is then applied to the B_SHG_ image (Fig. 5b) in order to evaluate the intensity in that area. This process is repeated through all the images acquired in the z-stack in order to measure the B_SHG_ signal throughout the thickness of the sample analyzed. In Fig. 5c the B_SHG_ intensity (measured as described above) of 3 MI samples is presented in comparison with 2 AMC samples as an example. Clearly an increased B_SHG_ intensity can be observed due to the increased fibrosis. But even more interestingly, a different spatial distribution through the sample thickness can be outlined. Control samples show a more superficial presence of collagen (the first 10 µm), while in the MI samples the B_SHG_ intensity increases also in central layers (see Video S1 and S2 for the entire z-stack and 3D rendering examples). To quantitatively evaluate the B_SHG_ intensity increase in the MI samples with respect to the controls the integral over 10 µm steps is performed and the mean and standard deviation is evaluated for the two groups. Fig. 5d shows the average intensity increase for each 10 µm step in the z-direction. All the controls analyzed showed a very similar distribution throughout all the thickness, while the MI samples show differences in collagen distribution along the z axis, reflected in the large standard deviation of the average intensity increase. On average, in the first superficial layers (20–30 µm) MI samples report nearly a 3-fold increase in B_SHG_ intensity (note that AMC samples show the presence of superficial collagen). More interestingly, at higher depth the B_SHG_ intensity ratio (MI/AMC) increases up to 6-fold, demonstrating the appearance of new collagen, not present in the AMC samples.

### Quantifying disease by Volume Analysis

**Figure 6:**
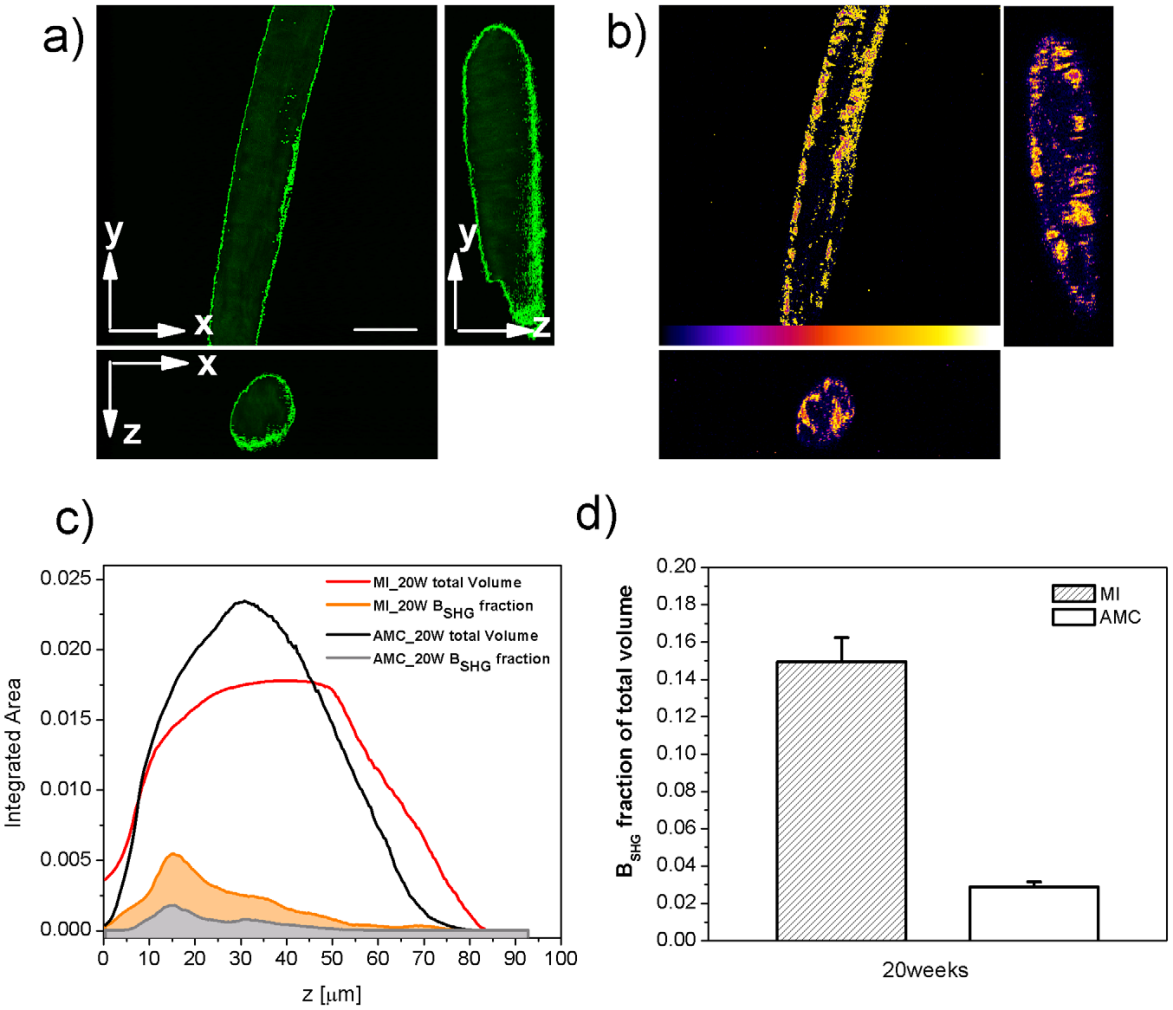
Volume Analysis. 3D (shown as xy view and orthogonal views xz, yz) autofluorescence a) and B_SHG_ b) from a MI sample. The region of interest selected by thresholding the autofluorescence to define the total volume of the sample is highlighted in green in a); the region of interest selected by thresholding the B_SHG_ to define the volume occupied by collagen is highlighted in yellow in b); c) the discretized area vs depth is plotted, black and red is from autofluorescence (AMC and MI samples respectively) grey and orange from B_SHG_ (AMC and MI sample respectively); the curves have been normalized so that the integral over black and red is 1, therefore representing the total volume, and over grey and orange is the fraction of volume occupied by collagen; d) average collagen presence evaluated for MI (n = 8) compared to AMC (n = 8) animals.

Finally we used autofluorescence and B_SHG_ to assess the volume of collagen in our samples. The autofluorescence image (Fig. 6a) is used to specify the selected tissue area in each optical slice (number of pixels). The B_SHG_ image is thresholded to count the number of pixels showing a B_SHG_ signal above background as illustrated in Fig. 6b. Repeating this process for each layer of the z-stack, a discretized volume for the sample and collagen is obtained from the autofluorescence and B_SHG_ images respectively. Fig. 6c shows the number of pixels evaluated for each z-layer (black and red lines from autofluorescence; orange and grey lines from B_SHG_): these data are normalized so that the area under the autofluorescence-related curves is equal to unity, hence representing the total volume of that sample, and the B_SHG_-related curves representing the percentage of collagen in that volume. Red and orange curves refer to MI samples; black and grey lines represent AMC samples. By integrating orange and grey curves the fraction of collagen present in that volume is then calculated. Fig. 6d shows the average collagen presence evaluated for the two groups MI and AMC (mean and st.dev from 8 animals each group). The MI samples show a 5-fold increase in volume occupied by collagen with respect to the AMC.

## Discussion

The aim of the present study is to measure and accurately quantify collagen accumulation, a feature of fibrosis induced by cardiac disease. In particular we study the example of cardiac disease in a rat model of chronic post myocardial infarction heart failure. Routine histology and immunohistochemistry are useful tools to identify the levels of increased fibrosis induced by cardiac disease, but they are inadequate to quantify the total collagen presence and the induced structural changes in fresh, untreated tissues. By contrast non-linear optical techniques as those used here present an attractive alternative to evaluate these morphological adaptations to disease *in vivo*. In particular, to quantitatively evaluate the collagen presence in the myocardium, a careful calibration of the acquisition settings is presented by performing measurements on rat tail samples and permeabilised skeletal muscle fibres as controls. In particular we show how to specifically separate SHG arising from collagen and SHG arising from myosin, by utilizing powers ranging from 10 to 20 mW (see Fig. 2i); we maximize the backward collection by controlling the solution ionic strength (see Fig. 3); and we minimize the effect of laser polarization by imaging rat samples in the same orientation. These calibrations are essential to quantitatively evaluate collagen presence within the myocardium. It's worth noting that by acquiring only the backward signal we are limited in quantifying the total collagen presence in absolute terms, particularly we may underestimate the collagen presence as there might be collagen fibrils whose backward signal is too low to be detected. This limit can be overcome by embedding within the tissue fluorescent beads which can be used as intensity normalization standards as fluorescence is isotropic thus not influenced by directionality. Still the method presented here provides comparative measurements and careful calibration of the system as shown here can yield quantitative results.

In the rat heart failure model, a 5-fold increase in collagen fibrosis is measured in the remote viable myocardium 20 weeks after infarction. Our results show a marked change in collagen distribution (see Fig. 5–6 and Video S1): AMC samples present, as expected, a thin extracellular superficial collagen sheath surrounding the left ventricular portion analyzed, while in the MI samples, collagen appears in between bundles of myocytes disrupting their precise networking organization. Specifically, elevated interstitial fibrosis, as shown here, may reduce tissue compliance and impair both contraction and relaxation [4]. Fibrosis also can disrupt conduction pathways and the patchy distribution may result in electrical heterogeneities which may be proarrhythmic [2]. These results confirm that an increase in collagen in tissue from the non-infarcted portion of rat hearts following myocardial infarction is an important part of the adverse cardiac remodelling that occurs in this disease. Although it is known that increased fibrosis is one of the reasons for the progressive decline in cardiac function post myocardial infarction, this novel technique allows assessment of this process quantitatively, in 3-D and with excellent spatial resolution. Besides the overall image quality improvement obtained thanks to the high specificity of the B_SGH_ light arising from collagen, which gives a better signal to noise ratio than routine histology methods, the 3D information gained by analyzing fresh, untreated tissue will be extremely useful to study myocardium adaptation to collagen increase. It is worth noting that all the measurements were carried out on trabecular preparations. Trabeculae should be representative of the LV wall's full thickness, but similar measurements in other regions of the myocardium should be performed. We used trabeculae as these represent the usual standards for mechanical measurements in order to couple the structural information obtained here with methods to probe the mechanical properties of the exact same samples. This combined approach will be unique in giving an insight into the structure-function relationship of the heart. It is particularly important to be able to visualize and quantify myocardial fibrosis, coupled to the mechanical response, in the burgeoning field of anti-fibrotic myocardial therapeutics [40], [41] where such quantification will be invaluable in the assessment of efficacy of the agents used.

Longer term we can foresee applicability of this technique beyond biomedical research and into clinical practice. The application of such a technique in conjunction with imaging endoscopes [42], [43] could open the door towards targeted local or systemic therapy for collagen deposition in heart failure.

## Supporting Information

Figure S1 **The dependence of B_SHG_ intensity on polarization has been characterized on rat tail samples by rotating the microscope stage in 10 degree steps.** The x-axis represents the angle between the main axis of the collagen fibre and the major axis of the laser polarization at the focus. A small intensity modulation (nearly 6%) is observed because the polarization status at the focus is elliptical and the tail samples presented fibrils at different orientation, thus flattening the response However, this result ensures that in our experimental conditions, the polarization direction doesn't affect the evaluation of the increase in B_SHG_ seen in MI samples compared to AMC.
(TIF)

Video S1 **Optical sectioning example of a MI sample showing B_SHG_ in fire pseudo scale colour and autofluorescence in green.**
(AVI)

Video S2 **3D rendering of the MI sample in movie1 showing B_SHG_ in fire pseudo scale colour and autofluorescence in green.**
(AVI)
