# Supplementary figures and images for: Non-Linear Optical Microscopy Sheds Light on Cardiovascular Disease
Source: PLoS One. 2013 Feb 7;8(2):e56136. doi: 10.1371/journal.pone.0056136 (PMC3567079; doi:10.1371/journal.pone.0056136)

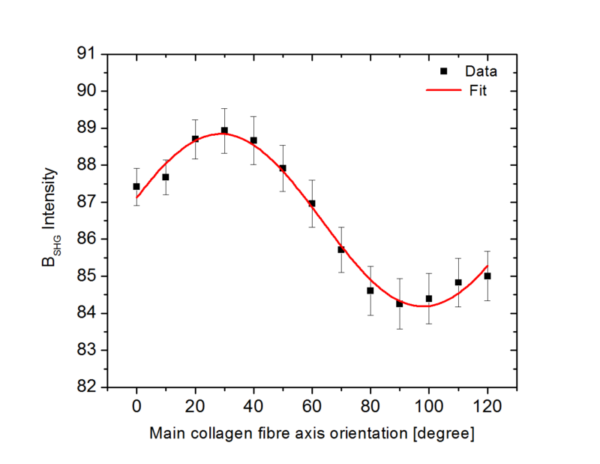

Supplement: Figure S1 — The dependence of BSHG intensity on polarization has been characterized on rat tail samples by rotating the microscope stage in 10 degree steps. The x-axis represents the angle between the main axis of the collagen fibre and the major axis of the laser polarization at the focus. A small intensity modulation (nearly 6%) is observed because the polarization status at the focus is elliptical and the tail samples presented fibrils at different orientation, thus flattening the response However, this result ensures that in our experimental conditions, the polarization direction doesn't affect the evaluation of the increase in BSHG seen in MI samples compared to AMC. (TIF) [file pone.0056136.s001.tif]
